# Whole-Genome Expression Analysis and Signal Pathway Screening of Synovium-Derived Mesenchymal Stromal Cells in Rheumatoid Arthritis

**DOI:** 10.1155/2016/1375031

**Published:** 2016-08-25

**Authors:** Jingyi Hou, Yi Ouyang, Haiquan Deng, Zhong Chen, Bin Song, Zhongyu Xie, Peng Wang, Jinteng Li, Weiping Li, Rui Yang

**Affiliations:** Department of Orthopedics, Sun Yat-sen Memorial Hospital, Sun Yat-sen University, 107 Yan Jiang Road West, Guangzhou 510120, China

## Abstract

Synovium-derived mesenchymal stromal cells (SMSCs) may play an important role in the pathogenesis of rheumatoid arthritis (RA) and show promise for therapeutic applications in RA. In this study, a whole-genome microarray analysis was used to detect differential gene expression in SMSCs from RA patients and healthy donors (HDs). Our results showed that there were 4828 differentially expressed genes in the RA group compared to the HD group; 3117 genes were upregulated, and 1711 genes were downregulated. A Gene Ontology analysis showed significantly enriched terms of differentially expressed genes in the biological process, cellular component, and molecular function domains. A Kyoto Encyclopedia of Genes and Genomes analysis showed that the MAPK signaling and rheumatoid arthritis pathways were upregulated and that the p53 signaling pathway was downregulated in RA SMSCs. Quantitative real-time polymerase chain reaction was applied to verify the expression variations of the partial genes mentioned above, and a western blot analysis was used to determine the expression levels of p53, p-JNK, p-ERK, and p-p38. Our study found that differentially expressed genes in the MAPK signaling, rheumatoid arthritis, and p53 signaling pathways may help to explain the pathogenic mechanism of RA and lead to therapeutic RA SMSC applications.

## 1. Introduction

Rheumatoid arthritis (RA) is considered a chronic, autoimmune joint disease characterized by systemic inflammation, autoantibodies, and synovitis with an unclear etiology [1]. Disease-modifying antirheumatic drugs (DMARDs) are conventional, key therapeutic agents that have been used to reduce systemic inflammation and synovitis [2]. When arthritis is uncontrolled or when DMARDs result in toxic effects, biological agents, for example, tumor necrosis factor (TNF) inhibitors and agents targeting the interleukin- (IL-) 1 and IL6 pathways, T-cell costimulatory pathways, and B cells will be used [3]. However, DMARDs do not effectively prevent cartilage damage, and the effects of biological agents remain largely unclear. Thus, it is necessary to develop new RA treatment methods.

Mesenchymal stromal cells (MSCs) are nonhematopoietic, multipotent stem cells that have been isolated and characterized from many human tissues, including bone marrow, synovium, adipose, and muscle [4]. As MSCs have an immunosuppressive property and are capable of multipotent differentiation, they have been widely applied for repairing tissue and treating immune disorders [5]. Synovium-derived mesenchymal stromal cells (SMSCs) not only have the immunosuppressive property and multipotent differentiation ability as other MSCs but also possess a greater ability for chondrogenesis [6], which could be very beneficial for repairing the cartilage damage caused by RA. Moreover, allogenic MSC transplantations could potentially be rejected or induce apoptosis in the allogeneic stem cells [7–9]; therefore, autologous SMSC treatments have massive potential for RA patients. However, the biological characteristics of RA SMSCs are largely unknown.

The aim of this study was to identify differentially expressed genes of SMSCs from the synovial tissue of RA patients and healthy donors (HDs). We found that there were 4828 differentially expressed genes in the RA SMSCs compared to the HD SMSCs. In addition, the mitogen-activated protein kinase (MAPK) signaling and rheumatoid arthritis pathways were both upregulated, and the p53 signaling pathway was downregulated. These findings could help explain the pathogenic mechanism of RA and potentially lead to RA SMSC-based therapeutic applications.

## 2. Materials and Methods

### 2.1. Cell Isolation and Culture

This study was approved by the ethics committee of Sun Yat-sen Memorial Hospital, and all study subjects provided written informed consent. Synovial tissue biopsies were obtained from 8 patients with RA and 6 HDs using 3.5 mm grasping biopsy forceps under arthroscopy. The RA patients fulfilled the American College of Rheumatology revised criteria for rheumatoid arthritis [10], and patients who were undergoing arthroscopy for meniscus injury without any autoimmune disease or any signs of osteoarthritis were regarded as HDs for practical purposes. The clinical status of each subject is shown in Table 1. The synovial tissue samples were rinsed three times with phosphate-buffered saline (PBS; Biovision). The samples were then finely minced and digested with 0.2% collagenase type II (MP) in high-glucose Dulbecco's modified Eagle's medium (HDMEM; GIBCO) containing 10% fetal bovine serum (FBS), 100 U/mL penicillin, and 100 U/mL streptomycin. After the samples were incubated overnight at 37°C, cells were collected by centrifugation, rinsed three times with PBS, resuspended in HDMEM, plated in a T25 culture flask, and allowed to attach for 3 days. Nonadherent cells were removed by changing the medium, and the attached cells were cultured in HDMEM at 37°C in a humidified 5% CO_2_ atmosphere until reaching confluence. The SMSCs were passaged upon reaching 80~90% confluence as previously described [11], and cells from passages 3–5 were used for experiments.

### 2.2. Immunophenotypic Characterization of SMSCs

The immunophenotypic characterization of SMSCs was performed by flow cytometry. After SMSCs were digested with 0.25% trypsin, the cells were resuspended with PBS in flow cytometry tubes. Aliquots of 5.0 × 10^5^ cells were incubated with conjugated monoclonal antibodies against CD34-PE, CD44-FITC, CD45-FITC, CD90-PE, CD105-PE, and HLA-DR-PE or conjugated isotype controls (all from BD Pharmingen) for 30 min in the dark. Flow cytometry was performed using a Becton Dickinson FACSCalibur, and the data were analyzed by CellQuest software (Becton Dickinson).

### 2.3. Multipotent Differentiation of SMSCs

Three previously described procedures were used for the multipotent differentiation of SMSCs [11]. For osteogenic differentiation, SMSCs were cultured for 21 days in HDMEM containing 0.1 *μ*M dexamethasone (Sigma), 50 *μ*M vitamin C (Sigma), 10 mM *β*-glycerophosphate (Sigma), 10% FBS, 100 U/mL penicillin, and 100 U/mL streptomycin and were then stained with alizarin red (Sigma). For chondrogenic differentiation, aliquots of 2.0 × 10^5^ cells were centrifuged at 500 ×g for 10 min; then, the cells were cultured for 21 days in centrifuge tubes with chondrogenic culture medium, which contained 6.25 *μ*g/mL insulin (Sigma), 6.25 *μ*g/mL transferrin (Sigma), 6.25 *μ*g/mL sodium selenite (Sigma), 1.25 *μ*g g/mL bovine serum albumin (BSA, Sigma), 1 mmol/L sodium pyruvate (Sigma), 37.5 *μ*g/mL vitamin C (Sigma), 50 ng/mL transforming growth factor-*β*_1_ (TGF-*β*_1_, Sigma), 2.5% FBS, 100 U/mL penicillin, and 100 U/mL streptomycin. After these cells were harvested, they were stained with toluidine blue (Sigma). For adipogenic differentiation, SMSCs were cultured for 21 days in HDMEM containing 1 *μ*M dexamethasone, 200 *μ*M indomethacin, 0.5 mM 3-isobutyl-1-methylxanthine, 10 *μ*g/mL insulin, 10% FBS, 100 U/mL penicillin, and 100 U/mL streptomycin and were then stained with oil red O (Sigma). All measurements were performed in triplicate. An inverted phase-contrast microscope (Nikon, Japan) was used to observe the cells and obtain images.

### 2.4. Total RNA Extraction and Quality Control

Total RNA was isolated from 8 RA and 6 HD SMSCs samples with 1 mL of TRIzol (Invitrogen) and was extracted using the phenol/chloroform method. Salt was washed away with 70% alcohol, and then total RNA was air-dried at room temperature. Total RNA was dissolved with an appropriate amount of RNase-free water. Standard denaturing agarose gel electrophoresis was used to assess RNA integrity, and a NanoDrop ND-100 was used to measure RNA quantity and quality.

### 2.5. RNA Labeling and Array Hybridization

Fourteen double-stranded cDNA (ds-cDNA) sequences were synthesized from the total RNA of 8 RA and 6 HD SMSC samples using an Invitrogen SuperScript ds-cDNA Synthesis kit. One *μ*g of ds-cDNA was labeled in the Human 12 × 135 K Gene Expression Array (Roche NimbleGen) with a NimbleGen One-Color DNA Labeling kit, according to the NimbleGen Gene Expression Analysis protocol (NimbleGen Systems, Inc.). Microarrays were hybridized with 4 *μ*g of Cy3-labeled ds-cDNA in NimbleGen Hybridization Buffer/Hybridization Component A in a hybridization chamber (Hybridization System-NimbleGen Systems, Inc.) at 42°C over 16 to 20 h. After hybridization, the 14 microarrays were washed in an ozone-free environment using a NimbleGen Wash Buffer kit (NimbleGen Systems, Inc.).

### 2.6. Microarray Scanning and Data Analysis

The fourteen microarrays were scanned using an Axon GenePix 4000B microarray scanner (Molecular Devices Corporation) piloted by GenePix Pro 6.0 software (Axon). For grid alignment and expression data analysis, the scanned images were then imported into the NimbleScan software, and the expression data were normalized using the Robust Multichip Average algorithm and quantile normalization. The probe-level and gene-level files were generated after normalization, and all gene-level files were further analyzed according to the RA and HD groupings using Agilent GeneSpring GX software (version 12.0). A scatter plot was used to visualize the data and access variation between RA and HD SMSCs. Volcano plot filtering was used to identify the genes differentially expressed between the two groups, and genes that exhibited greater than or equal to 2.0-fold change were selected for data analysis. Hierarchical clustering was also performed using Agilent GeneSpring GX software, and the variability between RA patient samples and HD samples was high while that in RA patient samples and HD samples was very low, which means that all these chips were available for further analysis. Then, gene symbols were uploaded to the Gene Ontology (GO, http://www.geneontology.org/) for GO annotation and enrichment analysis. The differentially expressed genes were used to perform pathway analyses in the most recent Kyoto Encyclopedia of Genes and Genomes (KEGG, http://www.genome.jp/kegg/) database.

### 2.7. Quantitative Real-Time Polymerase Chain Reaction (qRT-PCR)

Total RNA of the SMSCs was extracted and transcribed into cDNA as described. A LightCycler 480 Real-Time PCR System (Roche, Basel) was used to perform the qRT-PCR analyses with a SYBR Premix Ex Taq II kit (Takara, Otsu). The PCR primers are listed in Table 2. PCR amplification consisted of 95°C for 30 s, followed by 40 cycles of 95°C for 5 s and 60°C for 20 s. Specificity was verified by a melting curve analysis, and the relative amounts of target gene mRNA were normalized to the expression of the housekeeping gene GAPDH.

### 2.8. Western Blot

After being washed with ice-cold PBS three times, RA and HD SMSCs were lysed in 0.06 mL of cell lysis buffer (Beyotime) supplemented with a cocktail of protease inhibitors on ice for 30 min. The cells were centrifuged at 14,000 ×g for 30 min at 4°C, and then supernatants were collected. A BCA Protein Assay kit (CWBiotech) was used to measure the protein concentrations. After all the samples were boiled with 20% sample loading buffer (Beyotime), 20 *μ*L of each protein extract was electrophoresed using 10% sodium dodecylsulfate-polyacrylamide gel electrophoresis and then transferred to a polyvinylidene fluoride (PVDF) membrane (Millipore). The PVDF membranes were blocked in Tris-buffered saline with Tween-20 (Cell Signaling Technology) and 5% nonfat milk for 60 min at room temperature and then incubated overnight at 4°C with primary antibodies against GAPDH, p53, p-JNK, p-ERK, and p-p38 (dilution 1 : 1000; Cell Signaling Technology). The PVDF membranes were incubated with appropriate secondary antibodies (dilution 1 : 3000; Santa Cruz) for 60 min at room temperature. Then, protein expression levels were detected using enhanced chemiluminescence (Millipore) and quantified using ImageJ software (National Institutes of Health, USA).

### 2.9. Statistical Analysis

The results were analyzed using GraphPad Prism 6 (GraphPad Software, Inc.) and presented as the mean ± standard deviation (SD). Student's* t*-test was used to compare means between two groups.* P* values < 0.05 were considered statistically significant.

## 3. Results

### 3.1. Identification of SMSCs via Immunophenotype and Multipotent Differentiation In Vitro

**Figure 1:**
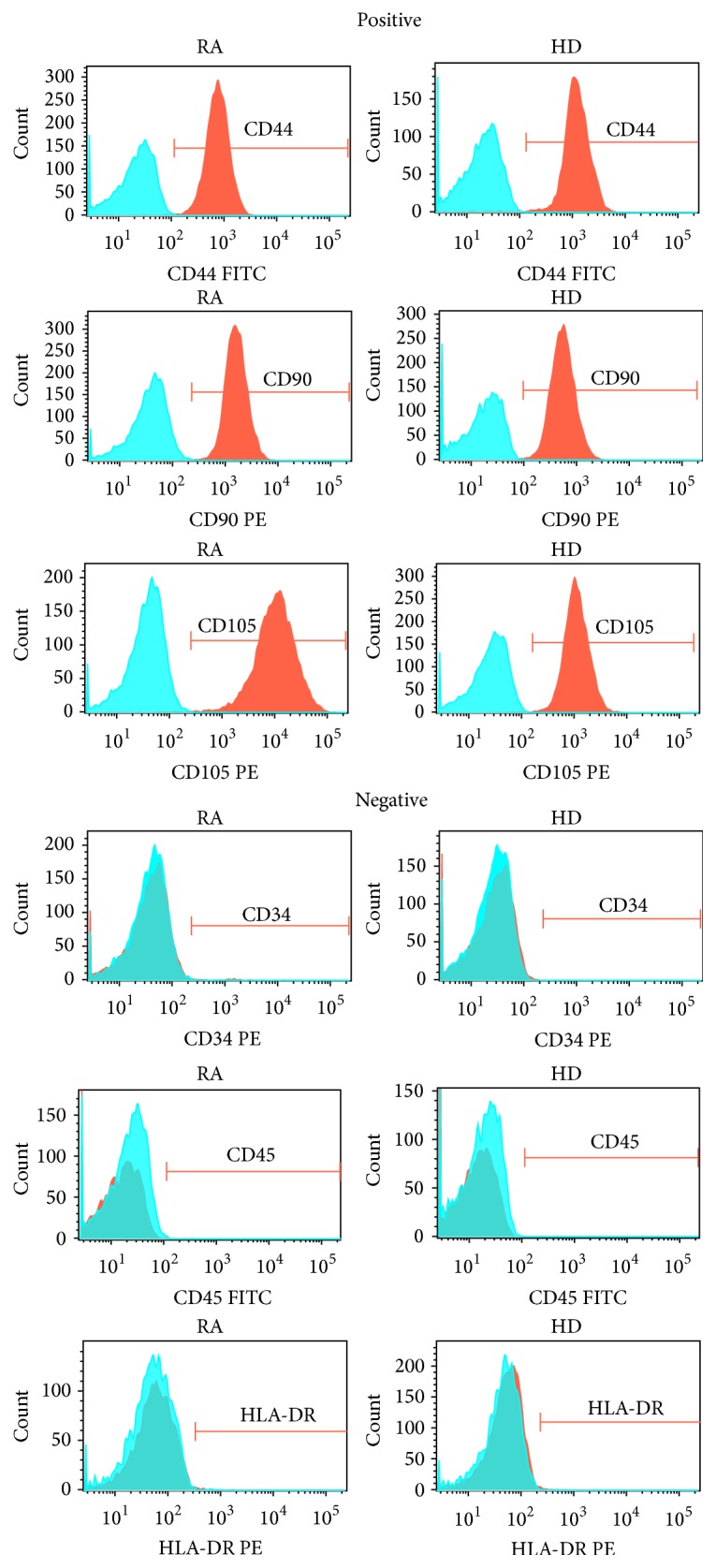
SMSC immunophenotypes. SMSCs from RA patients and HDs were positive for CD44, CD90, and CD105 and negative for CD34, CD45, and HLA-DR. Blue areas indicate background fluorescence of isotype control IgG, and red areas represent the fluorescence of the corresponding immunophenotype. The *x*-axis represents fluorescence intensity, and the *y*-axis represents cell count.

The immunophenotypic identification of SMSCs in the RA and HD groups was performed by flow cytometry. The cells in both groups were positive for CD44, CD90, and CD105 and negative for CD34, CD45, and HLA-DR, and no significant differences were found (*P* value > 0.05, Figure 1).

**Figure 2:**
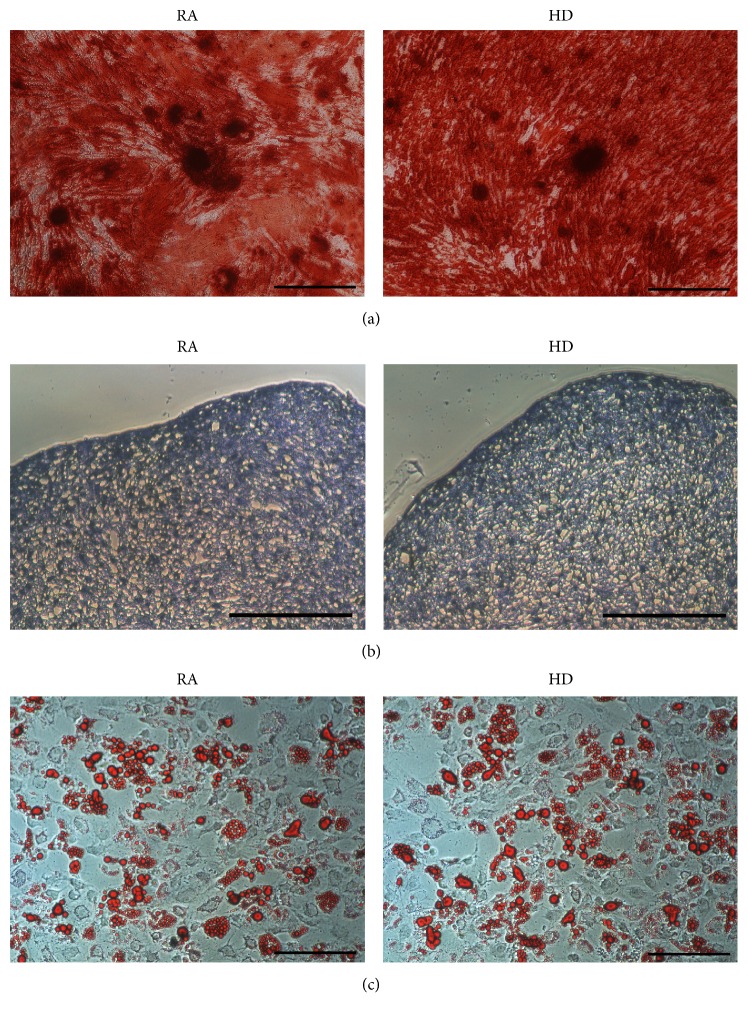
Triple SMSC differentiations. Osteogenic, chondrogenic, and adipogenic differentiation of SMSCs from RA patients and HDs were observed using an inverted phase-contrast microscope. (a) Osteogenic differentiation was indicated by mineralization nodes stained with alizarin red (scale bar: 200 *μ*m). (b) Chondrogenic differentiation was identified by glycosaminoglycans stained with toluidine blue (scale bar: 200 *μ*m). (c) Adipogenic differentiation was identified by lipid vacuoles stained with oil red O (scale bar: 200 *μ*m).

After induction for 21 d, the SMSCs had clearly differentiated into osteocytes, chondrocytes, and adipocytes. Osteogenesis was indicated by mineralization nodes that were observed after being stained with alizarin red (Figure 2(a)); chondrogenesis was indicated by glycosaminoglycans that were stained with toluidine blue (Figure 2(b)); and adipogenesis was indicated by lipid vacuoles that were stained with oil red O (Figure 2(c)). Together with the immunophenotypic identifications mentioned above, these results indicated that SMSCs from the RA and HD groups fulfilled the defining criteria for MSCs [12].

### 3.2. Scatter Plot and Volcano Plots

**Figure 3:**
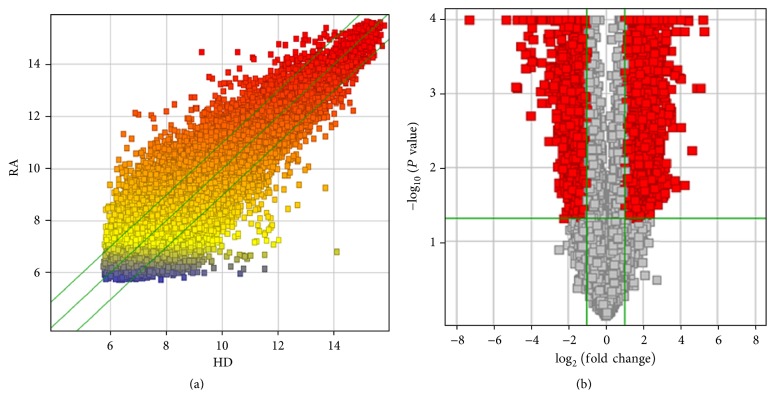
Scatter plot and volcano plots of differentially expressed genes. (a) Differentially expressed genes of SMSCs from both RA patients and HDs are shown in the scatter plot; each value represents a normalized signal value from each gene (log_2_ scale). Fold-change lines are shown as green lines in the scatter plot, and gene fold-changes ≥ 2.0 between the RA and HD groups are shown above the top green line and below the bottom green line. (b) The volcano plots were constructed using fold-change values (log_2_ scale) and *P* values (−log_10_ scale). The vertical lines correspond to 2.0-fold change up and down and the horizontal line represents a *P* value of 0.05. The red point in the plot represents the significantly differentially expressed genes.

All the data are shown in a scatter plot, which is a useful method for visualizing and assessing interchip variation (Figure 3(a)). Differential expression between RA and HD SMSCs was visualized using volcano plots (Figure 3(b)), which are constructed using fold-change values and *P* values and allow the visualization of the relationship between fold-change, that is, the magnitude of change, and statistical significance, which encompasses both the magnitude of change and variability.

### 3.3. GO Analysis

**Figure 4:**
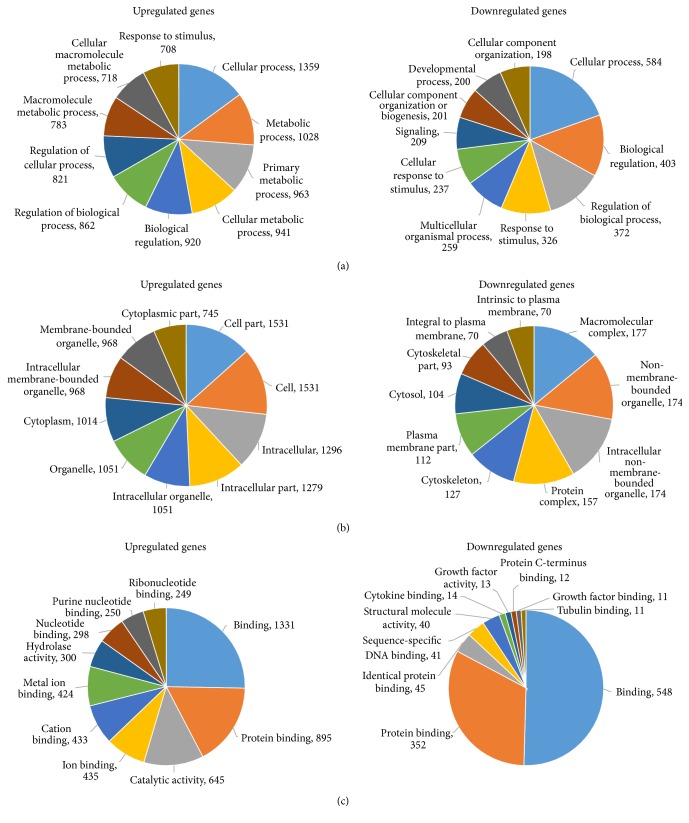
Gene Ontology (GO) analysis results of the genes differentially expressed in SMSCs from RA patients and HDs. Counts of the top ten significantly enriched terms of upregulated and downregulated genes in the biological process, cellular component, and molecular function domains are shown in (a), (b), and (c), respectively.

There were 4828 genes that were differentially expressed between the RA and HD groups (*P* < 0.05), of which 3117 were upregulated and 1711 were downregulated. GO was used to analyze the differentially expressed genes in three domains, that is, the biological process, cellular component, and molecular function domains. Counts of the top ten significantly enriched terms of the upregulated and downregulated genes are shown in Figure 4.

In the biological process domain, there were 797 significant functional description nodes in the upregulated genes and 380 significant functional description nodes in the downregulated genes. The top ten significantly enriched terms in the upregulated genes were cellular process, metabolic process, primary metabolic process, cellular metabolic process, biological regulation, regulation of biological process, regulation of cellular process, macromolecule metabolic process, cellular macromolecule metabolic process, and response to stimulus. The top ten significantly enriched terms in the downregulated genes were cellular process, biological regulation, regulation of biological process, response to stimulus, multicellular organismal process, cellular response to stimulus, signaling, cellular component organization or biogenesis, development process, and cellular component organization (Table 3).

In the cellular component domain, there were 125 significant functional description nodes in the upregulated genes and 52 significant functional description nodes in the downregulated genes. The top ten significantly enriched terms in the upregulated genes were cell part, cell, intracellular, intracellular part, intracellular organelle, organelle, cytoplasm, intracellular membrane-bounded organelle, membrane-bounded organelle, and cytoplasmic part. The top ten significantly enriched terms in the downregulated genes were macromolecular complex, non-membrane-bounded organelle, intracellular non-membrane-bounded organelle, protein complex, cytoskeleton, plasma membrane part, cytosol, cytoskeletal part, integral to plasma membrane, and intrinsic to plasma membrane (Table 4).

In the molecular function domain, there were 163 significant functional description nodes in the upregulated genes and 44 significant functional description nodes in the downregulated genes. The top ten significantly enriched terms in the upregulated genes were binding, protein binding, catalytic activity, ion binding, cation binding, metal ion binding, hydrolase activity, nucleotide binding, purine nucleotide binding, and ribonucleotide binding. The top ten significantly enriched terms in the downregulated genes were binding, protein binding, identical protein binding, sequence-specific DNA binding, structural molecule activity, cytokine binding, growth factor activity, protein C-terminus binding, growth factor binding, and tubulin binding (Table 5).

### 3.4. Screening of Signaling Pathways Related to RA SMSCs

**Figure 5:**
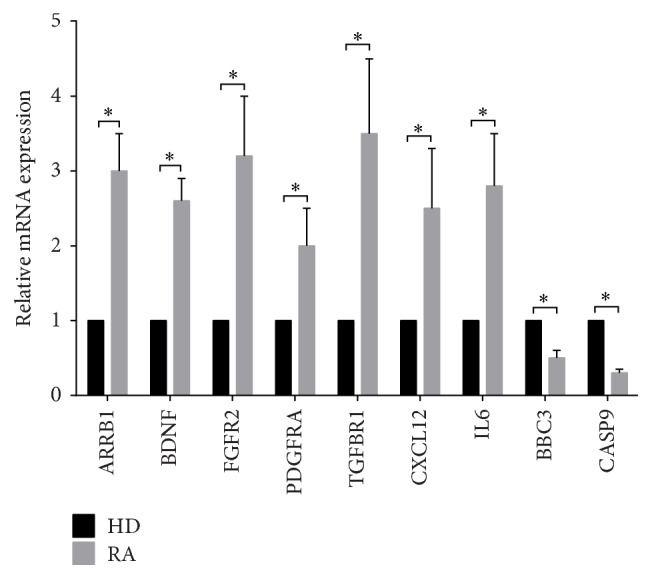
Quantitative real-time polymerase chain reaction (qRT-PCR) results of genes differentially expressed in SMSCs from RA patients and HDs. qRT-PCR was used to verify partial differentially expressed genes in the MAPK signaling, rheumatoid arthritis, and p53 pathways, including ARRB1, BDNF, FGR2, PDGFRA, TGFBR1, CXCL12, IL6, BBC3, and CASP9. The internal marker gene GAPDH was used for normalization. ^*∗*^ *P* < 0.05.

**Figure 6:**
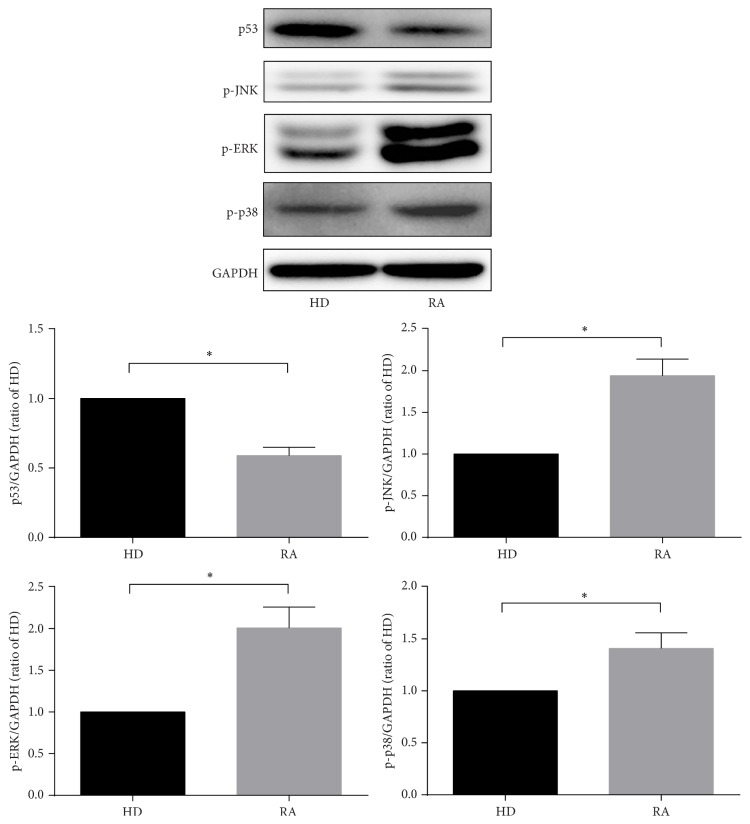
Western blot results of p53, p-JNK, p-ERK, and p-p38 expressed in SMSCs from RA patients and HDs. Western blot was used to determine the levels of p53 expression in the p53 pathway and p-JNK, p-ERK, and p-p38 expression in the MAPK signaling pathway. The internal marker protein GAPDH was used for normalization. ^*∗*^ *P* < 0.05.

Based on the most recent KEGG database, we carried out KEGG pathway analyses to determine the pivotal signaling pathways of the differentially expressed genes. The two highest enrichment scores for the upregulated genes in the RA group were hsa04010 (MAPK signaling pathway) and hsa05323 (rheumatoid arthritis pathway). Moreover, the highest enrichment score for the downregulated genes in the RA group was hsa04115 (p53 signaling pathway) (Table 5). In the MAPK signaling pathway, there were 47 differentially expressed genes: ARRB1, ATF2, BDNF, CACNA1C, CASP3, CHUK, CRK, DUSP10, DUSP3, DUSP5, EGFR, FGF10, FGF7, FGFR1, FGFR2, GNG12, HSPA2, HSPA6, HSPA8, IL1R1, JUN, MAP2K4, MAP3K12, MAP3K2, MAP3K4, MAP3K5, MAP3K7, MAP3K8, MAP4K3, MAP4K4, MAPK10, MAX, NF1, PDGFRA, PLA2G4A, PPM1A, PPP3CB, RAPGEF2, RPS6KA3, RRAS2, SOS1, SOS2, STK3, TGFB3, TGFBR1, TGFBR2, and ZAK (Table 6). In the rheumatoid arthritis pathway, 18 genes were expressed differentially, including ANGPT1, ATP6V0A1, ATP6V1A, ATP6V1C2, ATP6V1G1, CCL2, CTSK, CXCL1, CXCL12, CXCL5, CXCL6, HLA-DQA1, ICAM1, IL6, JUN, MMP1, MMP3, and TGFB3. In the p53 signaling pathway, there were 13 differentially expressed genes: BBC3, CASP9, CCNB1, CCNB2, GADD45B, GTSE1, IGF1, IGFBP3, RPRM, RRM2, SERPINE1, SESN2, and SESN3. The expression levels of several genes mentioned above were verified by qRT-PCR, including ARRB1, BDNF, FGFR2, PDGFRA, TGFBR1, CXCL12, IL6, BBC3, and CASP9 (Figure 5). Moreover, to further investigate the expression of p53 and MAPK signaling pathways, the expression levels of p53, p-JNK, p-ERK, and p-p38 were detected via western blot (Figure 6). Interestingly, the level of p53 expression was significantly lower and the levels of p-JNK, p-ERK, and p-P38 expression were significantly higher in RA SMSCs than in HD SMSCs. These findings further suggested that the MAPK pathway was upregulated and the p53 pathway was downregulated in RA SMSCs compared with HD SMSCs.

## 4. Discussion

In our previous work, we discovered that MSCs from patients with ankylosing spondylitis showed dysfunctional immunomodulation and differentiation, which may play an important role in the pathogenesis of ankylosing spondylitis or other chronic autoimmune diseases [13–16]. However, the immunomodulatory properties and differentiation capacities of RA SMSCs remain largely unknown. More than half of the risk of developing RA is due to genetic factors [17]. Nevertheless, except the PTPN22 and HLA genes, no major pathogenic genes have yet been identified to be associated with RA [1]. In this study, we explored the differences in whole-genome expression and signaling pathways of SMSCs from RA patients and HDs, which may lead to the discovery of the pathogenesis of RA. Our work identified 4828 differentially expressed genes in the RA group compared to the HD group, of which 3117 genes were upregulated and 1711 genes were downregulated.

The GO analysis showed that the genes with significant differences in the biological process domain were mostly related to cellular processes; in particular, upregulated genes were related to metabolic processes, and downregulated genes were related to biological regulation. In the cellular component domain, the upregulated genes were mainly related to intracellular organelles, particularly to the membrane-bounded organelles. Interestingly, intracellular non-membrane-bounded organelles accounted for the second largest proportion in downregulated genes. For the molecular function domain, the upregulated genes were mainly related to binding, including protein binding, ion binding, cation binding, metal ion binding, nucleotide binding, purine nucleotide binding, and ribonucleotide binding and catalytic activity, for example, hydrolase activity. Moreover, the downregulated genes were also mainly related to binding, especially protein binding.

The KEGG pathway analysis results showed that the MAPK signaling, rheumatoid arthritis, and p53 signaling pathways were differentially expressed in RA and HD SMSCs. The MAPK signaling pathway not only involves cytokine production and RA pathogenesis [18–20] but also regulates MSC differentiation, immunoregulation, and apoptosis [21–24], indicating that the MAPK signaling pathway may play an important role in RA SMSCs. We found 47 differentially expressed genes in the MAPK signaling pathway, some of which are involved in the immune system, inflammatory diseases, or MSC biology. For instance, high ARRB1 expression is related to the pathogenesis of experimental autoimmune encephalomyelitis and multiple sclerosis [25]. In addition, brain-derived neurotrophic factor (BDNF) has a neuroprotective role in experimental autoimmune encephalomyelitis and multiple sclerosis, which may have important implications for the corresponding therapies [26, 27]. Interestingly, BDNF expression has been reported to be higher in RA synovial tissue [28], and our results showed that BDNF expression was also higher in RA SMSCs, suggesting that BDNF expression in RA SMSCs is involved in the pathogenesis of RA. FGF-2 and Anosmin-1, which are markers for the level of inflammation of multiple sclerosis lesions, participate in oligodendrocyte precursor cell migration via FGF receptor 1 (FGFR1) [29]. Intrarenal mRNA levels of PDGFRA are significantly enriched in patients with lupus nephritis [30]. TGFBR1 can control the immunomodulatory properties of MSCs via regulating their IL6 and indoleamine 2,3-dioxygenase secretions [31]. In addition, FGFR1 [32], FGFR2 [33], CACNA1C [34], GNG12 [35], MAP3K8 [36], and Nf1 [37] are involved in the differentiation potential of MSCs, indicating that some differences in the differentiation capacities of RA and HD SMSCs might exist. Our results showed that the genes upregulated in RA SMSCs related to the MAPK signaling pathway may be broadly involved in both the pathogenesis of RA and the biology of RA SMSCs; however, further experiments should be conducted to explore the functions of these genes in RA SMSCs.

The JNK pathway, the ERK pathway, and the p38 pathway are three well-defined MAPK pathways, and the phosphorylation of JNK, ERK, and p38 can result in the catalytic activation of MAPK signaling [38]. The levels of p-JNK, p-ERK, and p-p38 expression were significantly higher in RA SMSCs than in HD SMSCs, further suggesting that the MAPK pathway was upregulated in RA SMSCs compared with HD SMSCs.

The rheumatoid arthritis pathway was also upregulated in RA SMSCs, with 18 differentially expressed genes. Proinflammatory cytokine-related genes, such as CCL2, CXCL1, CXCL5, CXCL6, CXCL12, and IL6, were upregulated in RA SMSCs. After stimulating CD4+ T cells and monocytes with CXCL12, osteoclast differentiation and T cell activation were enhanced via elevated expression levels of RANKL and TNF-*α* [39]. Upregulated CXCL12 expression in RA SMSCs may provide a helpful clue in elucidating the pathogenesis of and treating RA. IL6 is an important proinflammatory cytokine of RA that is involved in leukocyte activation, antibody production, and some systemic symptoms, such as asthenia, acute-phase mediator response, and anemia [40]. Moreover, IL6 has successfully been targeted in RA therapies [41]. Increased IL6 expression in RA SMSCs is helpful for demonstrating the important role of RA SMSCs in RA pathogenesis.

The p53 signaling pathway was downregulated in RA SMSCs, with 13 differentially expressed genes revealed by the whole-genome expression analysis. Moreover, the expression of the tumor suppressor p53, which has been reported to play an important role in mediating cellular stress responses and is involved in cell cycle arrest, metabolism, senescence, DNA repair, and importantly proapoptosis [42], was significantly lower in RA SMSCs compared to HD SMSCs. Downstream genes of p53, such as BBC3 [43], CASP9 [44], GADD45B [45], IGF1 [46], and IGFBP3 [47], have also been reported to play roles in certain proapoptotic effects. In our study, the above-mentioned genes were also downregulated in RA SMSCs, suggesting that RA SMSCs might have a stronger resistance to apoptosis. The apoptosis of transplanted MSCs has limited their therapeutic potential thus far [24]. As such, MSCs with a stronger resistance to apoptosis could demonstrate improved posttransplantation survival, which may be another advantage for autologous SMSC transplantation in RA patients.

## 5. Conclusion

In the present study, we screened differentially expressed genes of RA SMSCs and HD SMSCs and investigated them with GO and KEGG pathway analyses. We analyzed significantly enriched terms of differentially expressed genes in the biological process, cellular component, and molecular function domains. Moreover, we found that the MAPK signaling and rheumatoid arthritis pathways were upregulated and the p53 signaling pathway was downregulated in the RA SMSCs. All these data provide clues regarding the pathogenesis of RA and could facilitate therapeutic applications of RA SMSCs. Finally, future work will validate these differentially expressed genes and investigate both RA etiologies and potential therapeutic strategies.

## Figures and Tables

**Table 1:** Demographics and characteristics of rheumatoid arthritis (RA) patients and healthy donors (HDs).

Items	RA	HD
Number of patients	8	6
Sex		
Men	4	3
Women	4	3
Age (years)	45 (39–49)	43 (37–48)
Disease duration (years)	3.3 (0.3–5.8)	2.8 (0.1–4.8)
CRP (mg/L)	19.2 (6–58)	—
ESR (mm/h)	32 (10–73)	—

CRP: C-reactive protein; ESR: erythrocyte sedimentation rate.

**Table 2:** Primers used for quantitative real-time polymerase chain reaction.

Gene	Forward primer	Reverse primer
ARRB1	AAAGGGACCCGAGTGTTCAAG	CGTCACATAGACTCTCCGCT
BDNF	GGCTTGACATCATTGGCTGAC	CATTGGGCCGAACTTTCTGGT
FGFR2	AGCACCATACTGGACCAACAC	GGCAGCGAAACTTGACAGTG
PDGFRA	TGGCAGTACCCCATGTCTGAA	CCAAGACCGTCACAAAAAGGC
TGFBR1	GCTGTATTGCAGACTTAGGACTG	TTTTTGTTCCCACTCTGTGGTT
CXCL12	ATTCTCAACACTCCAAACTGTGC	ACTTTAGCTTCGGGTCAATGC
IL6	ACTCACCTCTTCAGAACGAATTG	CCATCTTTGGAAGGTTCAGGTTG
BBC3	GACCTCAACGCACAGTACGAG	AGGAGTCCCATGATGAGATTGT
CASP9	CTTCGTTTCTGCGAACTAACAGG	GCACCACTGGGGTAAGGTTT

**Table 3:** GO analysis of RA SMSCs with different biological processes related to differentially expressed genes.

GO ID	Term	Regulation	Number of genes	Percentage of total gene number	Fold enrichment	*P* value
GO:0009987	Cellular process	Up	1359	43.60%	1.078949506	3.45058*E* − 16
GO:0008152	Metabolic process	Up	1028	32.98%	1.127180781	2.26411*E* − 11
GO:0044238	Primary metabolic process	up	963	30.90%	1.163226478	3.42172*E* − 14
GO:0044237	Cellular metabolic process	Up	941	30.19%	1.157274694	8.30096*E* − 13
GO:0065007	Biological regulation	Up	920	29.52%	1.134286715	9.96280*E* − 10
GO:0050789	Regulation of biological process	Up	862	27.65%	1.130113501	2.91311*E* − 08
GO:0050794	Regulation of cellular process	Up	821	26.34%	1.133035445	7.34418*E* − 08
GO:0043170	Cellular macromolecule metabolic process	Up	718	23.03%	1.162237783	1.77085*E* − 08
GO:0050896	Response to stimulus	Up	708	22.71%	1.108798503	7.11907*E* − 05
GO:0006807	Nitrogen compound metabolic process	Up	568	18.22%	1.063792239	0.028072195
GO:0009987	Cellular process	Down	584	34.13%	1.030034922	0.031637828
GO:0065007	Biological regulation	Down	403	23.55%	1.103818079	0.001406004
GO:0050789	Regulation of biological process	Down	372	21.74%	1.083465722	0.012748648
GO:0050896	Response to stimulus	Down	326	19.05%	1.134212455	0.001202522
GO:0032501	Multicellular organismal process	Down	259	15.14%	1.094089824	0.036294594
GO:0051716	Cellular response to stimulus	Down	237	13.85%	1.208258943	0.000275304
GO:0023052	Signaling	Down	209	12.22%	1.142323863	0.012379382
GO:0071840	Cellular component organization or biogenesis	Down	201	11.75%	1.275102033	4.79255*E* − 05
GO:0032502	Developmental process	Down	200	11.69%	1.192861477	0.002106076
GO:0016043	Cellular component organization	Down	198	11.57%	1.295981777	2.02658*E* − 05

**Table 4:** GO analysis of RA SMSCs with different cellular components related to differentially expressed genes.

GO ID	Term	Regulation	Number of genes	Percentage of total gene number	Fold enrichment	*P* value
GO:0005623	Cell	Up	1531	49.12%	1.027156657	2.40220*E* − 05
GO:0005622	Intracellular	Up	1296	41.58%	1.143609988	4.14799*E* − 22
GO:0043229	Intracellular organelle	Up	1051	33.72%	1.120831397	8.22135*E* − 10
GO:0005737	Cytoplasm	Up	1014	32.53%	1.277346130	3.84962*E* − 31
GO:0043231	Intracellular membrane-bounded organelle	Up	968	31.06%	1.147230634	3.86604*E* − 11
GO:0044444	Cytoplasmic part	Up	745	23.90%	1.315817744	3.25995*E* − 22
GO:0044446	Intracellular organelle part	Up	607	19.47%	1.163042085	1.40257*E* − 06
GO:0005634	Nucleus	Up	565	18.13%	1.073142161	0.017245693
GO:0032991	Macromolecular complex	Up	361	11.58%	1.081921665	0.042183953
GO:0043234	Protein complex	Up	314	10.07%	1.127231548	0.008327837
GO:0032991	Macromolecular complex	Down	177	10.34%	1.18550075	0.006000202
GO:0043232	Intracellular non-membrane-bounded organelle	Down	174	10.17%	1.521121372	2.70923*E* − 09
GO:0043234	Protein complex	Down	157	9.18%	1.259572268	0.000893262
GO:0005856	Cytoskeleton	Down	127	7.42%	1.939965930	1.27657*E* − 13
GO:0044459	Plasma membrane part	Down	112	6.55%	1.210497238	0.017123710
GO:0005829	Cytosol	Down	104	6.08%	1.201689499	0.025595975
GO:0005887	Integral to plasma membrane	Down	70	4.09%	1.334366755	0.007950984
GO:0031226	Intrinsic to plasma membrane	Down	70	4.09%	1.309313339	0.011994087
GO:0015630	Microtubule cytoskeleton	Down	61	3.57%	2.133880899	1.80251*E* − 08
GO:0005694	Chromosome	Down	41	2.40%	1.75267224	0.000358288

**Table 5:** GO analysis of RA SMSCs with different molecular functions related to differentially expressed genes.

GO ID	Term	Regulation	Number of genes	Percentage of total gene number	Fold enrichment	*P* value
GO:0005488	Binding	Up	1331	42.70%	1.107406186	6.17161*E* − 19
GO:0005515	Protein binding	Up	895	28.71%	1.259276446	1.94840*E* − 23
GO:0003824	Catalytic activity	Up	645	20.69%	1.218132345	6.46805*E* − 11
GO:0043167	Ion binding	Up	435	13.96%	1.110490713	0.004421943
GO:0043169	Cation binding	Up	433	13.89%	1.108515605	0.005140801
GO:0046872	Metal ion binding	Up	424	13.60%	1.097341066	0.011174111
GO:0016787	Hydrolase activity	Up	300	9.62%	1.291345373	4.92602*E* − 07
GO:0000166	Nucleotide binding	Up	298	9.56%	1.314022658	1.04955*E* − 07
GO:0017076	Purine nucleotide binding	Up	250	8.02%	1.329326120	5.67347*E* − 07
GO:0032553	Ribonucleotide binding	Up	249	7.99%	1.332560002	4.92004*E* − 07
GO:0005488	Binding	Down	548	32.03%	1.037520988	0.031108598
GO:0005515	Protein binding	Down	352	20.57%	1.127010990	0.001007060
GO:0042802	Identical protein binding	Down	45	2.63%	1.385306122	0.016715118
GO:0043565	Sequence-specific DNA binding	Down	41	2.40%	1.388762475	0.020934665
GO:0005198	Structural molecule activity	Down	40	2.34%	1.491048874	0.007638802
GO:0019955	Cytokine binding	Down	14	0.82%	2.778713450	0.000496933
GO:0008083	Growth factor activity	Down	13	0.76%	1.793577236	0.030091084
GO:0008022	Protein C-terminus binding	Down	12	0.70%	1.810133333	0.034106543
GO:0019838	Growth factor binding	Down	11	0.64%	2.262666667	0.009349359
GO:0015631	Tubulin binding	Down	11	0.64%	2.183274854	0.012057826

**Table 6:** KEGG pathway analysis results for RA SMSCs.

Pathway ID	Definition	Regulation	Selection counts	Gene names	*P* value
hsa04010	MAPK signaling pathway	Up	47	ARRB1, ATF2, BDNF, CACNA1C, CASP3, CHUK, CRK, DUSP10, DUSP3, DUSP5, EGFR, FGF10, FGF7, FGFR1, FGFR2, GNG12, HSPA2, HSPA6, HSPA8, IL1R1, JUN, MAP2K4, MAP3K12, MAP3K2, MAP3K4, MAP3K5, MAP3K7, MAP3K8, MAP4K3, MAP4K4, MAPK10, MAX, NF1, PDGFRA, PLA2G4A, PPM1A, PPP3CB, RAPGEF2, RPS6KA3, RRAS2, SOS1, SOS2, STK3, TGFB3, TGFBR1, TGFBR2, and ZAK	0.002378379
hsa05323	Rheumatoid arthritis	Up	18	ANGPT1, ATP6V0A1, ATP6V1A, ATP6V1C2, ATP6V1G1, CCL2, CTSK, CXCL1, CXCL12, CXCL5, CXCL6, HLA-DQA1, ICAM1, IL6, JUN, MMP1, MMP3, and TGFB3	0.018097090
hsa04115	p53 signaling pathway	Down	13	BBC3, CASP9, CCNB1, CCNB2, GADD45B, GTSE1, IGF1, IGFBP3, RPRM, RRM2, SERPINE1, SESN2, and SESN3	0.000017539
